# Consistent Failure to Produce a Cognitive Load Effect in Visual Working Memory Using a Standard Dual-Task Procedure

**DOI:** 10.5334/joc.108

**Published:** 2020-07-10

**Authors:** Timothy J. Ricker, Evie Vergauwe

**Affiliations:** 1 College of Staten Island, City University of New York, US; 2 University of Geneva, CH

**Keywords:** working memory, short-term memory, visual memory, multitasking, cognitive load, forgetting

## Abstract

Working memory performance is impaired when an attention-demanding task is executed during memory retention. The cognitive load effect is the consistent finding that the size of the memory impairment is determined by the relative amount of time that the secondary processing task occupies attention during memory retention. Cognitive load has been proposed to be a Priority-A benchmark any model of working memory should be able to explain (Oberauer et al., 2018), in part because the effect appears to generalize across different experimental procedures and materials. Using a standard dual-task procedure, we detail four experiments using a visual working memory recall task, two requiring memory for low-level features and two requiring memory for canonical angles (up, down, left, right, etc.). In all four experiments, we failed to find a cognitive load effect, calling into question the generality of the cognitive load effect and whether it is driving forgetting in multitasking contexts.

The working memory system maintains information in an immediately available state for ongoing cognition. Much recent work has argued that concurrent processing demands of a task, or cognitive load, drive forgetting from working memory (Barrouillet, Bernardin, & Camos, 2004; Barrouillet, Bernardin, Portrat, Vergauwe, & Camos, 2007; Barrouillet & Camos, 2015; Barrouillet, Portrat, & Camos, 2011; Bayliss, Bogdanovs, & Jarrold, 2015; Camos, Lagner, & Barrouillet, 2009; Hudjetz & Oberauer, 2007; Liefooghe, Barrouillet, Vandierendonck, & Camos, 2008; Vergauwe, Barrouillet, & Camos, 2010). Cognitive load is a measure of attentional demand that indexes how much maintenance can occur during memory retention. Attention is thought to maintain memory representations in the absence of verbal rehearsal through a process called refreshing (Barrouillet et al., 2004; Camos et al., 2018; Cowan, 1992; Raye, Johnson, Mitchell, Greene, & Johnson, 2007; Vergauwe & Cowan, 2014). When attention is needed for a concurrent task, it is not available for refreshing and, as a result, forgetting occurs.

## An important role for cognitive load in working memory

Cognitive load is often defined as the proportion of time attention is occupied by a secondary task during memory retention, which can be increased by, for example, increasing the pace at which a secondary task needs to be performed (Barrouillet et al., 2004; Barrouillet et al., 2007; Barrouillet & Camos, 2015; Barrouillet et al., 2011; Hudjetz & Oberauer, 2007). This conception follows from the assumptions of the Time-Based Resource-Sharing (TBRS) model proposed by Barrouillet et al. (2004) who assumed that unattended working memory representations are forgotten with the passage of time. In order to keep memories from becoming inactive and forgotten, attention must be directed toward each representation, in sequence (i.e., refreshing). Critically, the TBRS model assumes that any secondary task that requires attention will prevent refreshing from occurring at the same time. Higher cognitive loads mean less time is available for memory maintenance and more time for trace decay to occur. Thus, the cognitive load of the secondary task has an inverse relationship to the total number of items that can be maintained in working memory. There are several alternative models of cognitive load effects with somewhat different assumptions, but all of them predict a strong, inverse relationship between cognitive load and the total number of items remembered in dual-task situations (Lemaire & Portrat, 2018; Oberauer & Lewandowsky, 2011; Oberauer, Lewandowsky, Farrell, Jarrold, & Greaves, 2012; Portrat & Lemaire, 2015). Accordingly, the cognitive load effect has been proposed to be a Priority-A benchmark any model of working memory should be able to explain (Oberauer et al., 2018), in part because the effect appears to generalize across different experimental procedures and materials.

The notion that forgetting is always entirely determined by cognitive load was, however, questioned by Ricker and Cowan (2010) and Vergauwe, Camos, and Barrouillet (2014) who raised the possibility that memory representations of detailed sensory features cannot be maintained by attention-based mechanisms and, therefore, are forgotten gradually across the absolute length of a retention interval. This previous work suggests that a pre-existing conceptual representation may be necessary for attention-based refreshing to function. Critically, under this assumption, one must predict that memory materials predominantly composed of low-level perceptual features do not show cognitive load effects. In the experiments that follow, we tested the prediction that memory items predominantly composed of low-level perceptual features will not show a cognitive load effect, whereas memory items predominantly composed of conceptual representations will show a cognitive load effect.

## Experiment 1

In our first experiment we test the prediction that materials predominantly composed of low-level sensory features lacking a conceptual long-term memory representation do not produce cognitive load effects. If attention cannot be used to maintain such representations, then memory performance for these stimuli should not suffer from increased cognitive load of the secondary task.

### Method

#### Participants

One-hundred and six students (67 female, ages 18–41)^1^ from universities in the United States participated in the experiment in exchange for partial course credit. Ten participants were excluded from the analysis because they did not complete the experiment.

In cognitive load experiments, the failure to observe a cognitive load effect could be due to the secondary task not inducing a load or because participants fail to perform the secondary task. In order to ensure that the secondary task was performed diligently, we only include trials with perfect secondary task accuracy in our analysis. This resulted in five participants without any data for analysis in one or more experimental conditions who were removed from the analyses reported in the results section (see Table 1 for results with alternative filtering). The inferential results reported in the text are reported in column 3 of Table 1 (in bold font) for all experiments. This left forty-three participants included in the analysis of Experiment 1a and forty-eight participants included in the analysis of Experiment 1b.

**Table 1:** Performance Metrics across Experiments and Inclusion Criteria.

Participant Inclusion Criteria	.8 secondary task accuracy or better	.8 secondary task accuracy or better	Must have a high accuracy* trial in all conditions	All participants that completed the study

Trial Inclusion Criteria	High accuracy* trials only	All trials	High accuracy* trials only	All trials

Experiment 1a (continuous memoranda + tone task)				
Participant N	38	38	**43**	44
Effect Size (d)	0.11	0.42	**0.07**	0.46
Bayes Factor (H_10_)	0.33	0.80	**0.12**	3.94
Experiment 1b (continuous memoranda + parity task)				
Participant N	30	30	**48**	52
Effect Size (d)	0.13	0.50	**0.12**	0.43
Bayes Factor (H_10_)	0.12	1.05	**0.08**	3.89
Experiment 2a (canonical memoranda + tone task)				
Participant N	14	14	**22**	30
Effect Size (d)	–0.34	0.19	**0.01**	0.28
Bayes Factor (H_10_)	0.31	0.14	**0.07**	0.19
Experiment 2b (canonical memoranda + parity task)				
Participant N	24	24	**37**	53
Effect Size (d)	0.24	0.48	**0.24**	0.47
Bayes Factor (H_10_)	0.24	0.70	**0.29**	14.82

*Note*: Effect sizes and Bayes factors pertain to the effect of Cognitive Load. * A high accuracy trial is one with perfect performance on the secondary task.

#### Materials

**Figure 1:**
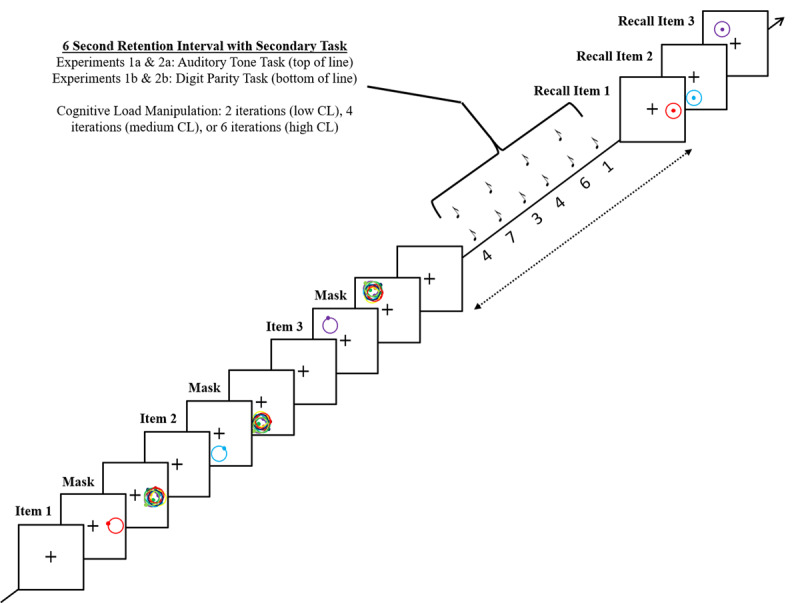
An example of a single experimental trial in Experiment 1.

The memory items were rings with a dot placed randomly along the outer circumference (Figure 1). The location of the dot on the edge of the circle was determined randomly on each trial, ranging anywhere within 90 degrees to the left or right of the top of the circle (Experiment 1a) or across the entire range of the circle (Experiment 1b). Each stimulus had the following properties in Experiments 1a & 1b respectively: ring diameter 2.5/2.3 cm, dot diameter 0.4/0.3 cm, ring center located at one of eight locations 4.5/4.2 cm from the center of the screen. The 8 locations were the location directly above the center of the screen and the 7 remaining locations that resulted from 45 degrees steps around a circle.

The response probe consisted of presentation of the memory stimulus ring in its original color, size, and location but with the dot located in the center of the ring. Participants were instructed to move the dot to the location in which they saw it during study.

In Experiment 1a participants heard a series of tones during our secondary task. Each individual tone was presented for 250 ms at a high (523.252 Hz) or low (130.813 Hz) pitch. In Experiment 1b participants saw a series of numbers during the secondary task. The numbers ranged from 1 to 8 and were presented visually at the center of the screen in 30-point font. Each number was presented until a response was made or until the next stimulus was presented. Two different secondary tasks were used to assess the robustness of our findings. See the design section for the presentation schedule in each cognitive load condition.

#### Design

We used a within-subjects design in which all non-fixed variables, such as item location, the angle of the dot location, and the cognitive load, were determined randomly with an equal probability on each trial. There were 3 possible levels of cognitive load, our variable of interest, resulting in a one-factor design with 3 levels. In Experiment 1a, one two-choice tone judgement was to be performed every 3 seconds, every 1.5 seconds, or every second, for the low, medium, and high load conditions respectively. The retention interval was always 6s. Experiment 1b used an identical design, except that the two-alternative choice task was a parity judgement of a presented number instead of a tone judgment task. There were 8 practice trials in Experiment 1a and 12 practice trials in Experiment 1b, followed by either 4 or 5 blocks of 30 experimental trials in both experiments.

#### Procedure

Except for the nature of the secondary task, all aspects of the procedure were identical between Experiments 1a and 1b, see Figure 1 for a single experimental trial. Participants pressed the space bar to begin each trial, which began with a white fixation cross presented at the center of a black screen for 500 ms. Next came item presentation, with each item being presented one at a time and followed by a post-perceptual mask and a consolidation period before presentation of the next item or, in the case of the final item, before the retention interval began. Each item was displayed for 400 ms, followed by a post-perceptual mask displayed for 200 ms. The mask was presented at the same location as the memory item and consisted of 8 circles, each slightly displaced from the location of item presentation and eight dots randomly placed in the region of item presentation. Next, there was a 200 ms blank screen during which only the fixation cross was presented.

After all three memory items were presented the 6s retention interval began. The first stimulus in the secondary cognitive-load task was presented at the time of retention interval onset. In Experiment 1a participants heard a series of tones and it was the participant’s task to push the down arrow key if the tone was low pitched or the up-arrow key if the tone was high pitched. In Experiment 1b participants saw a series of numbers presented in the center of the screen. It was the participant’s task to push the ‘a’ key if the number was odd or the ‘s’ key if the number was even. The level of cognitive load for the current trial determined the rate and number of secondary-task stimulus.

After the retention interval, memory for each of the three items was tested in the order of item presentation. For each item, a probe ring was presented in the location of the original memory item with a dot in its center. Participants were to move the dot to the original location of the memory item. Feedback was given for all three memory items simultaneously by displaying the correct location of each dot and the participant’s entered response. Sound feedback was also given during this period. In Experiment 1a, if the average error on the present trial was less than 12 degrees, an increasing-frequency happy sound sequence was played. If average error was between 12 and 40 degrees a slightly increasing frequency neutral sound sequence was played. If the average error was greater than 40 degrees, a decreasing frequency sad sound sequence was played. In Experiment 1b, the feedback tone thresholds were shifted to 20 and 60 degrees respectively to account for the larger stimulus presentation space when using the entire circle.

### Results

**Figure 2:**
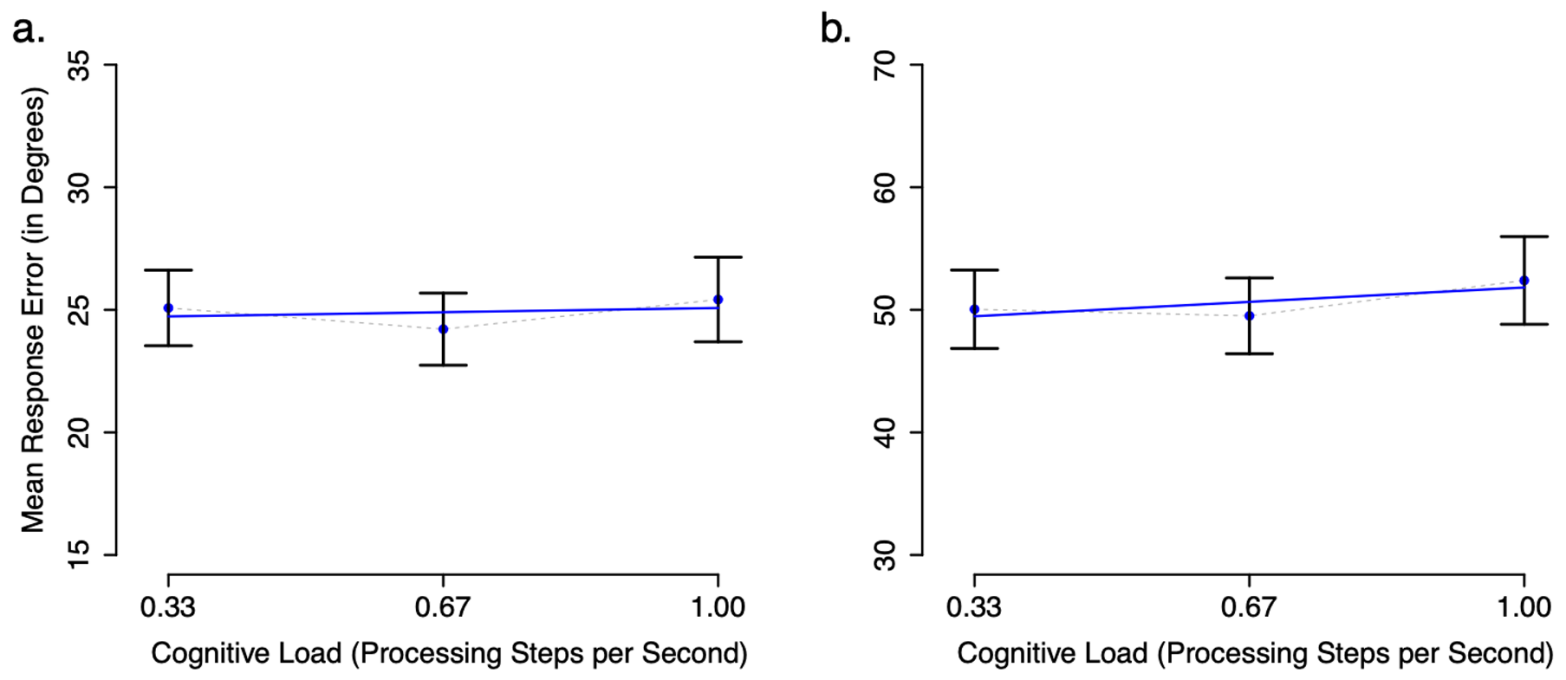
Mean response error in degrees of angle by cognitive load condition (number of digits/6s retention interval) observed in Experiment 1a (continuous memoranda + tone task; panel **a**) and Experiment 1b (continuous memoranda + parity task; panel **b**) Error bars represent standard error of the mean. The blue line shows the linear regression of mean response error on cognitive load. Note that the y-axis is compressed in panel a. relative to panel b. due to Experiment 1a having one half the total stimulus range compared to Experiment 1b.

Visual examination of Figure 2 shows no effect of Cognitive Load on mean response error in Experiment 1a or 1b. Mean error was overall larger in Experiment 1b than in 1a, which was to be expected given that the stimulus space was larger in Experiment 1b (see the materials section for stimulus details). Repeated-Measures ANOVAs of mean response error as a function of Cognitive Load confirms the lack of a cognitive load effect in Experiment 1a, *F*(2,84) = 1.16, d_z_ = 0.07, Bayes factor = 8.62 in favor of the null (means: 0.33 = 25, 0.67 = 24, 1.00 = 25), and in Experiment 1b, *F*(2,94) = 0.82, d_z_ = 0.12, Bayes factor = 12.83 in favor of the null (means: 0.33 = 50, 0.67 = 50, 1.00 = 52). The Bayes factors were calculated following the method described by (Rouder, Morey, Speckman, & Province, 2012) using the BayesFactor package for R (Morey & Rouder, 2015) with the standard deviation of the effect size set to (√2)/2.

### Discussion

Experiments 1a and 1b give clear evidence against an effect of cognitive load on memory performance when remembering materials that are predominantly composed of continuous low-level perceptual features. As can be seen in Table 1, using other types of data filtering to assess the robustness of our findings show that some modest evidence for an effect of cognitive load can be found, but only when no filtering was applied to the data, which has, to our knowledge, never been done. Cognitive load studies typically exclude participants and/or trials with poor secondary-task performance. Note that we have used cognitive load manipulations that correspond to three different pace levels that have frequently been used in previous cognitive load studies (e.g., Barrouillet et al., 2007; Vergauwe et al., 2010; Vergauwe, Hartstra, Barrouillet, & Brass, 2015).

## Experiment 2

**Figure 3:**
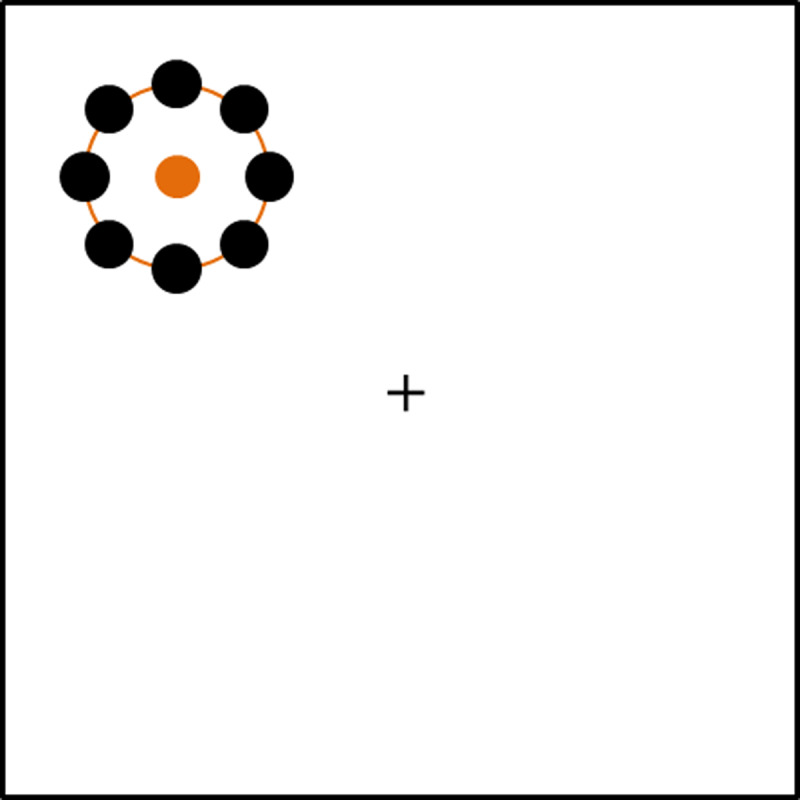
An example of a response probe in Experiment 2b. The black dots show the eight possible dot locations for canonical memory items. Each memory item only contained a single dot.

In Experiment 2 we explore the same set of cognitive load conditions as in Experiment 1, but with canonical memory stimuli instead of the continuous memory stimuli. Experiments 2a and 2b are the same as Experiments 1a and 1b, except that the location of the dots on the rings could now only appear at the four cardinal directions, up, down, left, right, and the 4 locations that lie halfway between these locations (see Figure 3 for an example). In Experiment 2b we also marked the possible presentation locations of the categorical items of the probe during memory recall. We expected to find a cognitive load effect for these memoranda, given that all past research indicates that this should be the case with categorical memoranda (Barrouillet et al., 2004; Barrouillet et al., 2007; Barrouillet & Camos, 2015; Barrouillet et al., 2011; Bayliss et al., 2015; Camos et al., 2009; Hudjetz & Oberauer, 2007; Liefooghe et al., 2008; Vergauwe et al., 2010).

### Method

#### Participants

Ninety students (53 female, ages 18–43) from the College of Staten Island participated in exchange for partial course credit. Seven participants were excluded from the analysis because they did not complete the experiment. After filtering out trials that did not show perfect secondary task performance, twenty-four participants did not have any data in one or more experimental conditions, leading to their exclusion from the sample. This left twenty-two participants included in the analysis of Experiment 2a and thirty-seven participants included in the analysis of Experiment 2b.

#### Materials

All materials were the same as in Experiment 1b, with the following exceptions. In Experiment 2 all memory stimuli were canonical locations on the ring, with the dot located at the top of the ring or one of the 7 other locations resulting from moving 45 degrees around the edge of the ring (see Figure 3). In Experiment 2b the memory-probe ring also marked all possible presentation locations with a white circle at each of the 8 locations. The secondary task stimuli in Experiments 2a and 2b were the same as those in Experiments 1a and 1b, respectively.

#### Design

All aspects of the design were the same as in Experiment 1, except that Experiments 2a and 2b had 4 blocks of 30 experimental trials.

#### Procedure

Except for the canonical nature of the memory stimuli in Experiment 2, all aspects of the procedure were the same in Experiment 2 as in Experiment 1. In Experiment 2b the canonical nature of the stimuli was repeatedly stressed in the instructions and practice sections of the experiment. Response markers were also placed on the response probe to mark the possible stimulus locations in Experiment 2b.

### Results

**Figure 4:**
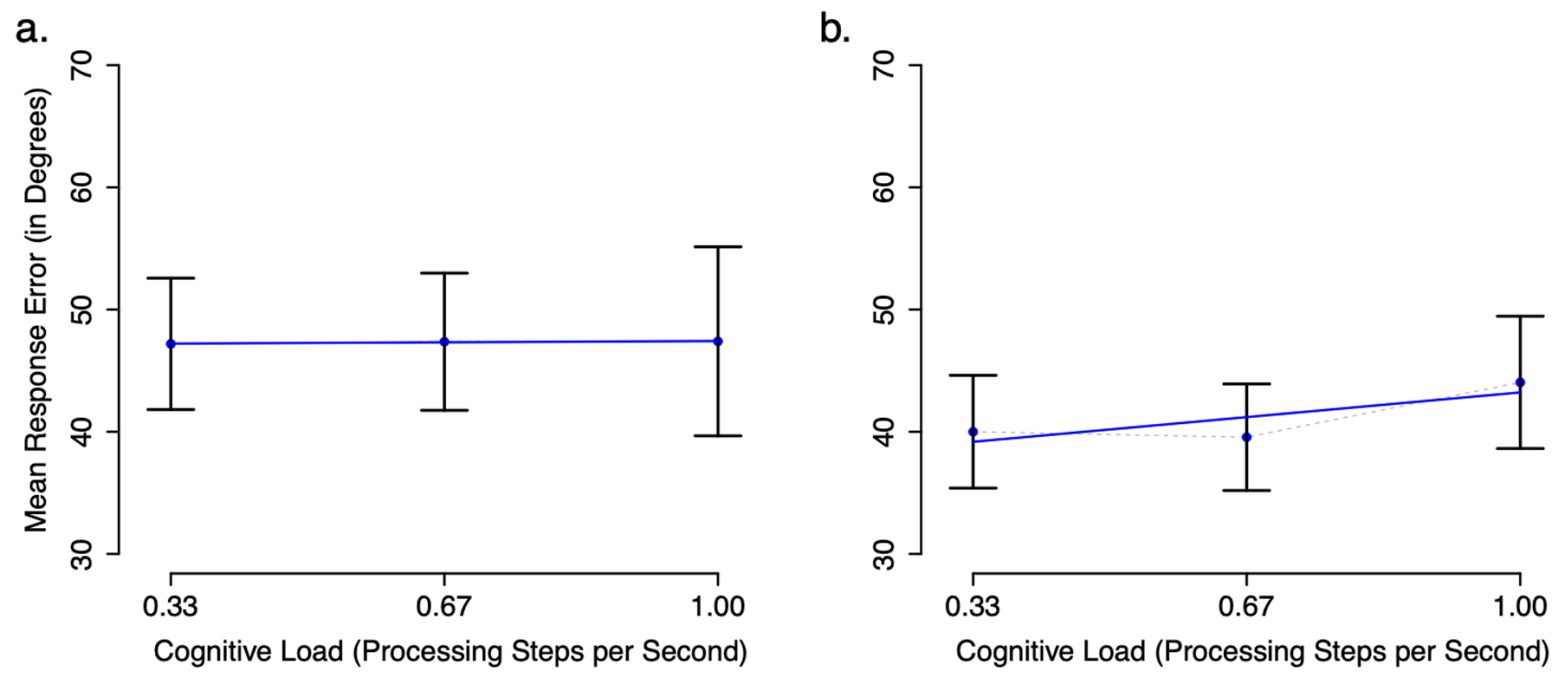
Mean response error in degrees of angle by cognitive load condition (number of digits/6s retention interval) observed in Experiment 2a (canonical memoranda + tone task; panel **a**) and experiment 2b (canonical memoranda + parity task; panel **b**) Error bars represent standard error of the mean. The blue lines show the linear regression of mean response error on cognitive load.

Visual examination of Figure 4a shows no effect of Cognitive Load on mean response error in Experiment 2a and the slightest hint of an effect in Experiment 2b. Repeated-Measures ANOVA of mean response error as a function of Cognitive Load confirms this lack of an effect for Experiment 2a, *F*(2,42) = 0.001, d_z_ = 0.01, Bayes factor = 13.68 in favor of the null (means: 0.33 = 47, 0.67 = 47, 1.00 = 47), and indicate no effect in Experiment 2b, *F*(2,72) = 2.12, d_z_ = 0.24, Bayes factor = 3.42 in favor of the null (means: 0.33 = 40, 0.67 = 40, 1.00 = 44).

### Discussion

The results of Experiment 2 demonstrate no effect of cognitive load on the recall of canonical visual memory items. As can be seen in Table 1, when no filtering whatsoever was applied to the data, there was still evidence against an effect of Cognitive load in Experiment 2a, whereas there was evidence for an effect of Cognitive load in Experiment 2b.

## General Discussion

We hypothesized that low-level features cannot be maintained using directed attention, whereas conceptual representations can. Therefore, we predicted no cognitive load effect for low-level features used in Experiment 1, whereas we did expect a cognitive load effect for conceptual memory materials used in Experiment 2. Despite the contrast in memory materials between the two experiments, the results were highly similar; neither experiment produced an effect of cognitive load. This is a novel finding. More research will be needed to explore why we have failed to find an effect of cognitive load in our two experiments despite consistent findings in favor of similar cognitive load effects for visuo-spatial memoranda in the published literature (e.g., Barrouillet, De Paepe, & Langerock, 2012; Langerock, Vergauwe, & Barrouillet, 2014; Vergauwe, Barrouillet, & Camos, 2009; Vergauwe et al., 2010; Vergauwe, Dewaele, Langerock, & Barrouillet, 2012; Vergauwe et al., 2015). Below, we speculate on four potential reasons.

First, one clear difference between our experiments and most of the previous work examining cognitive load effects is our use of a Brown-Peterson task. In the Brown-Peterson task all of the memory items are presented before a single retention interval, during which a series of secondary task executions is required (Brown, 1958; Jarrold, Tam, Baddeley, & Harvey, 2011; Lucidi et al., 2016; Neath, VanWormer, Bireta, & Surprenant, 2014; Peterson & Peterson, 1959). Past work on cognitive load effects is largely, though not uniquely, built on the complex-span task (Barrouillet et al., 2004; Barrouillet et al., 2007; Oberauer et al., 2012; Vergauwe et al., 2009, 2010). In this latter task there is a retention interval after each memory item, with a series of secondary task executions during each retention interval. From existing theoretical perspectives there is no reason that cognitive load effects should require multiple individual retention intervals after each item, but perhaps something about multiple retention intervals contributes to cognitive load effects.

Second, the presentation duration may have played a role in our failure to observe a cognitive load effect by allowing considerable time for working memory consolidation. Working memory consolidation is the process by which a fragile sensory memory trace is stabilized into working memory and made resistant to forgetting (Chun & Potter, 1995; Jolicœur & Dell’Acqua, 1998; Ricker, 2015; Ricker & Cowan, 2014). De Schrijver and Barrouillet (2017) systematically varied a period of free time between item presentation and secondary task onset during which attention could be used to focus on the memory item for consolidation. When this consolidation period was longer, they observed smaller cognitive load effects on memory performance. Perhaps the present failure to observe a cognitive load effect stems from allowing relatively long consolidation times for our memory items.

Third, the finding could be specific to the stimuli used in our study. To our knowledge, the present study is the first to use an angle reproduction task to explore cognitive load effects. Fourth, perhaps some combination of these previous explanations is responsible for our failure to observe cognitive load effects.

To conclude, the current findings are surprising and in contradiction with a large amount of past research showing the ubiquitous nature of cognitive load effects (Barrouillet et al., 2004; Barrouillet et al., 2007; Barrouillet et al., 2011; Bayliss et al., 2015; Hudjetz & Oberauer, 2007; Oberauer et al., 2018; Vergauwe et al., 2009, 2010). While more research is needed to understand the key factor in determining the presence vs. absence of cognitive load effects, our results show that there are clear unexpected boundary conditions to the effect.

## Data Accessibility Statement

All data reported in this work are available on the Open Science Framework at https://osf.io/w9mv6/.

## Footnotes

Six of the participants in Experiment 1a did not complete the experiment and the program failed to record their demographic data as a result. The gender and age breakdowns omit these participants. Only participants that completed the full experiment are included in the data analysis.
